# 

**Published:** 1875-08

**Authors:** 


### TABLE OF CONTENTS.

ORIGINAL COMMUNICATIONS.

Acute Articular Rheumatism. By C. H. Tidd, M.D., Middleport, O.. .... ...449
Retention and Suppression of Urine. By R. J. Preston, M.D., Virginia ....459
Medical Organization. By A. A. Lyon, M.D., Shreveport, La. .............462
Letter from New York........................................466

SELECTIONS.

On Iodoform as a Remedy in the Treatment of Affections of the Cornea and Conjunctiva ........................................469
Treatment of Eczema ........................................472
Treatment of Erysipelas ........................................475
Radical Cure for Hernia........................................476
On the Prevention of Mammary Abscesses by the Application of the Principle of Rest........................................478

EXTRACTS AND GLEANINGS.

The Curative Treatment of Insanity by Chlorhydrate of Morphia—482. Bromide of Potassium in Strychnine-Poisoning—483. Phosphide of Zinc—484. On Hydrocele of the Neck—485. Coxalgia successfully Treated by the Use of Sayre's Hip-Joint Splint—486 Phosphorous in Neuralgia; Surgery of Arteries—487. Acidulated Gargles in Typhoid Fever—488. Critique on the Esmarch Method; Removal of Foreign Bodies from the Throat by Injection—489. A Pathognamonic Symptom of the Moribund Condition; Opium or Salicin in Diarrhœa—490. A New Antiseptic Dressing; Ovulation without Menstruation—491. Browbeating Hysteria; Enemata of Bromide of Potassium in Obstinate Vomiting—492. Ergot as an Anti-galactagogue; Eucalyptus and Tobacco—493. Administration of Purgatives by the Hypodermic Method; Charcot on the Relief of Hysterical Seizures by Compression of the Ovaries; Solution of Camphor in Erysipelas—494. Prevention of the Formation of Milk; A Remarkable Instance of Scarlatinal Contagion; Local Use of Tannin—495. Salicylic Acid in Diphtheria; A Male Girl; Acute Hematuria Treated Succesefully by Local Applications—496. Phosphorous as a Stimulant; Iodine as a Substitute for Iodide of Potassium; To Prevent the Marks of Small-Pox—497. Treatment of Acute and Chronic Bronchitis and Asthma; Delirium in Typhoid Fever; Warts—498. Absolute Repose in Tetanus; Insecticides—499. Use of Quinine in the Treatment of Infantile Diseases; Xonthium Strumarium; Cough and Sweating in Phthisis—500. Salicylic Acid; Longevity in England; Solution of Hypodermic Injection—501. The First Authentic Satire on Physicians; Boston Medical Library—502. A Certain Sign of Death; Goa Powder in Skin Diseases; Sterility—503.

EDITORIAL AND MISCELLANEOUS.

To Our Readers; The Delay of the Record........................................504

From Dr. G. W. ........................................505

Medical Colleges ........................................508

Dr. T. E. A.; Dr. J. C. D. ........................................509

Our Advertisers........................................510

Communications on Hand; Deaths........................................511

Medical Association of Alabama........................................512

